# Epstein-barr virus detection in nasopharyngeal carcinoma-implications in a low-risk area

**DOI:** 10.1590/S1808-86942010000300007

**Published:** 2015-10-20

**Authors:** Eduardo Breda, Raquel Jorge Ferreira Catarino, Isabel Azevedo, Marisa Lobão, Eurico Monteiro, Rui Medeiros

**Affiliations:** 1 MD, ENT; 2 MSc, Researcher, Microbiologist; 3 MD, Oncologist; 4 MD, Radiologist; 5 MD, ENT; 6 PhD, Researcher, Pharmacist. Instituto Português de Oncologia Francisco Gentil do Porto, EPE

**Keywords:** herpesvirus 4 human, epstein-barr virus infections, nasopharyngeal neoplasms

## Abstract

Several studies have been published concerning Epstein-barr virus (EBV) infection and nasopharyngeal cancer (NPC) development. The incidences of histological types are different according to endemic or non-endemic regions. Latent EBV infection is found in almost all cases of NPC in endemic regions, but normally absent in type I carcinomas, more common in non-endemic regions.

**Aim**: The purpose of this hospital-based study was to analyze the presence of EBV in nasopharyngeal tumor tissues and in peripheral blood of nasopharyngeal cancer patients and healthy individuals, in a low risk, non-endemic area.

**Methods**: EBV detection in samples of nasopharyngeal cancer patients and healthy individuals.

**Results**: This study indicates that the frequency of EBV positive cases in peripheral blood is higher in advanced tumor stages.

**Conclusions**: The incidence rates of NPC have a distinct distribution. Since the prevalence of this disease is low in occidental countries, little is known about the biology of these tumors in non-endemic areas. We observed statistically significant differences in EBV detection between the NPC patient group and the control group. This study may help to understand the biological mechanisms of NPC and the correlation of EBV infection with this disease, in a low risk, non-endemic region.

## INTRODUCTION

The nasopharyngeal carcinoma (NPC) has different geographic and ethnic distribution when compared to other head and neck tumors. In 2000 there were 64,798 cases reported throughout the world and over 80% of these came from Asian countries, especially China and other southeastern Asian countries^1^. Globally speaking it is a rare disease, with an incidence below 1/100,000 in Caucasian individuals from North America and Western countries. The highest incidence is found in Southern China (25-30/100,000 individuals per year), especially in Canton.

NPC is a different type of head and neck cancrum. The World Health Organization (WHO) considers three histological types of NPC based on differentiation grade. Type I concerns keratinizing epidermoid carcinomas; Type II are the non-keratinizing and Type III are the undifferentiated carcinomas, also called lymphoepitheliomas – characterized by a prominent lymphocytic infiltrate. This interaction between tumor cells and lymphocytes seems crucial for the continuous spread of the Type III^2^ malignant carcinoma components. Of these, variants II and III are the most frequent and have common etiological characteristics, associated with the Epstein – Barr virus (EBV) infection. First described by Epstein and Barr in 1964^3^, the EBV is a Gamma Herpes virus of the lymphocryptovirus genus, taxonomically called Human Herpes Virus 4, and man is its natural and only host.

The primary infection is usually acquired during childhood and 95% of the adults have the virus in their latent form within the host's B cells. The virus is the agent responsible for infectious mononucleosis and its associations with the Burkitt Lymphoma and NPC are well known^4^. The endemicity rate for the Epstein-Barr virus varies according to the geographic region investigated, being very high in Northern Africa (Algeria and Tunisia) and China, and very low in Northern Europe (Denmark and Holland)^5^.

Nasopharyngeal tumors are made up of neoplastic cells derived from the non-keratinized epithelium with an inflammatory infiltrate in the stroma and have a bad prognosis among malignant head and neck tumors. Studies in the literature show that the EBV infection in nasopharyngeal epithelial cells happen before clonal expansion of the tumor cells population6. Studies in normal nasopharyngeal tissues and on biopsies of pre-malignant tissues show the presence of genetic alterations at an early stage of carcinogenesis, indicating that the stable infection of EBV epithelial cells requires a changed cell environment^5^.

Many studies have been published concerning the association between EBV infection and nasopharyngeal carcinogenesis development7. The prevalence of histology types are different comparing endemic and non-endemic regions. In endemic areas, Type III represents over 97% of the cases, while the keratinizing type is more common in western countries (∼75%)^2^. Beyond histological differences, the latent infection by the Epstein-Barr virus is present in almost all NPC cases in endemic regions, but it is usually not present in Type I carcinomas – more common in non-endemic regions^8^.

A hospital-based study previously published by our group showed that contrary to what has been published by other western countries, the prevalence of Types II and III in Northern Portugal was higher than in the other groups, with 93.75% of non-keratinizing carcinomas and only 6.25% of keratinizing carcinomas, in a total of 350 patients analyzed^9^.

The objective of the present study was to detect the Epstein-Barr virus in nasopharyngeal tumor tissue and peripheral blood from patients with nasopharyngeal carcinoma and healthy individuals coming from a non-endemic low-risk area.

## MATERIALS AND METHODS

### Patient selection

We reviewed the cases of nasopharyngeal carcinoma that came to the ENT Department of our Institute.

We studied 43 cases with fragments included in paraffin blocks, from which we found EBV in 19 cases of undifferentiated carcinoma of the nasopharynx (10 men and 9 women) (ages between 13 and 86 years). We also included in the study 17 nasopharyngeal tumor tissue biopsies and samples of the peripheral blood from 32 patients with undifferentiated carcinoma of the nasopharynx (ages between 20 and 71 years) and 45 samples from the blood donors without known oncologic disease with ages between 18 and 64 years).

### DNA extraction and purification

The viral DNA in the paraffin bloc samples was extracted by means of an enzymatic digestion using 200ml of digestion buffer (TrisHCL 10mM, KCl 50mM, MgCl2 2.5mM, 0.5% Tween 20). The tubes were incubated during the night at 37°C, and the lysis was interrupted by incubation at 95°C during 10 min. The DNA obtained was purified by the phenol-chloroform method and resuspended in 50ml of bi-distilled water. The samples were frozen at −20°C for later use.

In regards of the peripheral blood samples, we collected 5ml of peripheral blood from each patient into a tube with EDTA in order to isolate the plasma. The samples were centrifuged at 1600 x g and the plasma was carefully removed from the EDTA tubes and transferred to polypropylene tubes. They were stored at −20°C until processed. The plasma DNA samples were extracted by means of the Qiagen Blood mini kit (Qiagen). The protocol utilized followed the steps recommended by the manufacturer. We used 200 μL of plasma to extract the DNA through columns.

DNA extraction from nasopharyngeal biopsy samples was carried out from fresh tissue, using the QIAamp Tissue Kit (Qiagen), following the protocol recommended by the manufacturer.

EBV was analyzed in the tumoral tissue from samples in paraffin blocks. Viral DNA amplification was carried out through the PCR nested method using the following primers (DE 5′ to 3′): E3-44mer: GCGGGTG-GAGGGAAAGG; E5-25mer: GTCAGCCAAGGGACGCG; E3-2PCR: GCCACCTGGCAGCCCTAAAG and E5-2PCR: AGGCTGCCCACCCTGAGGAT. The E3-44mer and E5-25mer primers were utilized in the first PCR reaction, being followed by another PCR reaction with the E3-2PCR and E5-2PCR primers, and the final product weighed 184pb. The PCR reactions were used in a programmable thermocycler (Biometra), bringing to each PCR tube 2ml of DNA to the following mixture: buffer 1X, 1.5mM of MgCl2, 0.2mM of dNTPs, 0.5mM of each primer and 1U of Taq polymerase. The first PCR reaction conditions were as follows: initial denaturation at 94°C during 5min; 40 cycles of 30s at 94°C; 30s at 57°C and 1min at 72°C, and one step of final extension of 7min at 72°C. Two ml of the product from the first reaction were subjected to a second PCR. The second reaction parameters were identical to those of the first one, except for the annealing temperature, which went from 57°C to 50°C. We did an agarose gel electrophoresis at 3% (w/v) dyed in ethidium bromide and studied under ultraviolet light.

### EBV analysis in the tumoral tissue of biopsies and peripheral blood

EBV detection in the fresh tissue and peripheral blood samples was carried out through the PCR technology in real time. The PCR primers were selected in a way as to pair them to the BALF5 region of the viral genome which codes the viral polymerase DNA. The forward and reverse primer sequences were, respectively: 5′- CGGAAGCCCTCTGGACTTC- 3′ and 5′- CCCTGTTTA-TCCGATGGAATG – 3′. A fluorogenic probe was used (VIC5′- TGTACACGCACGAGAAATGCGCC – 3′TAMRA) with a sequence between the PCR primers, synthesized by PE Applied Biosystems (Foster city, Calif.). The PCR reaction was done using the PCR Taq-Man (PE Applied Biosystems) kit. In short: 2.5 μL of DNA extraction solution from 200 μL of plasma added to a PCR mixture with de plasma 10 mM of Tris (pH 8.3), 50 mM of KCl, 10 mM of EDTA, 5 mM of MgCl2, 100 μM of dATP, dCTP, dGTP and dTTP, 0.2 μM of each primer, 0.1 μM of the probe and 1.25 U of AmpliTaq Gold (PE Applied Biosystems). Amplification followed, starting with a 50°C cycle during 2min., AmpliTaq Gold activation during 10 min. at 95°C, 47 cycles of 15 sec. at 95°C and 1 minute at 62°C. The entire process was done by an ABIPRISM 7300 (PE Applied Biosystems) sequence detector.

### DNA Quality Control

We did the PCR for the ß-globin gene sequence aiming at confirming the presence of an amplifiable DNA. The target sequence was a 110pb segment and we used the following primers (from 5′ to 3′): PCO3: ACACAAC-TGTGTTCACTAGC; PCO4: CAACTTCATCCACGTTCACC. The protocol included forty 20s cycles at 95°C, 45s at 55°C and 45s at 75°C.

### Statistical analysis

The statistical analysis of the results was done using the SPSS (vs. 15.0) statistical software. Differences among mean values were calculated using the t-Student test. The Chi-Squared test was used to compare the frequencies of the categorical variables. A value of p<0.05 was considered statistically significant.

## RESULTS

The sample description in the group of cases with nasopharyngeal carcinoma (NPC) and in the control group is depicted on Table 1. In the group of cases with nasopharyngeal carcinoma we used the samples extracted from the nasopharyngeal tumoral tissue in paraffin blocks and the fresh tissue and peripheral blood of diseased individuals. In the control group, made up of individuals without known oncologic disease, we used samples from the peripheral blood. We studied a total of 68 individuals with NPC and 45 healthy controls. Among the cases, 19 samples came from tumoral tissue in paraffin blocks (mean age of 56.6 years, standard deviation of 11.4), 17 samples from fresh tissue (mean age of 51.8 years, 7.4 of standard deviation) and 32 samples from peripheral blood (mean age of 48.1 years, standard deviation of 11.3) from NPC patients. The mean age of control group individuals was 51.8 years (standard deviation of 7.4). The frequencies of males and females were: 73.5% and 26.5%, respectively in the group of individuals with NPC and 60.0% and 40.0%, respectively in the control group.

**Table 1:** Sample description in the group of cases of nasopharyngeal carcinoma of the nasopharynx and in the control group.

	Patients with Nasopharyngeal Carcinoma			Control Group
		Type of sample		
	Paraffin blocs	Fresh tissue biopsy	Periphery blood	Peripheral blood
Number of cases		17	32	45
Mean age ± standard deviation	56.6 ± 11.4	51.8 ± 7.4	48.1 ± 11.3	51.8 ± 7.4
Gender				
Males	10 (52.6%)	13 (76.5 %)	27 (84.4%)	27 (60.0%)
Females	9 (27.4%)	4 (23.5 %)	5 (15.6%)	18 (40.0%)

The frequency of positive EBV cases in the tumoral tissue samples in paraffin blocks was 84.2%. The results of EBV detection in the fresh tissue samples showed a 100% rate of positive cases, corroborating other studies published which show that all the nasopharyngeal tumor cells had viral DNA10, 11. In the cases of samples from the peripheral blood of NPC patients we found 53.1% of positive EBV cases. In the control group we did not find EBV DNA in the peripheral blood of healthy individuals (Table 2). We found statistically significant differences in the positive and negative EBV cases in the control group and in the group of patients with NPC (P<0.001) in all types of samples analyzed; Table 2).

**Table 2:** EBV detection result in the control group and in the group of patients with nasopharyngeal carcinoma according to the type of sample.

	EBV detection		p value*
	Positive (%)	Negative (%)	
Control group			
Peripheral blood (n=45)	0 (0.0)	45 (100.0)	–
Nasopharyngeal carcinoma cases			
Paraffin blocks (n=19)	16 (84.2)	3 (15.8)	<0.0001
Fresh tissue biopsy (n=17)	17 (100.0)	0 (0.0)	<0.0001
Peripheral blood (n=32)	17 (53.1)	15 (46.9)	<0.0001
Total (n=68)	50 (73.5)	18 (26.5)	<0.0001

* Chi-squared test

Table 3 depicts the results from comparing the EBV positive and negative cases insofar as the gender of patients with NPC and controls were concerned. We did not find statistically significant differences in the distribution of positive and negative EBV cases as to the gender of the groups analyzed. The analysis of EBV detection according to tumor stage (Table 4) indicates that the frequency of positive EBV cases is higher in more advanced tumors, despite the fact that such difference is in the very threshold of statistical significance (p=0.069).

**Table 3:** Comparison of the positive and negative EBV results frequencies concerning the gender of patients with nasopharyngeal carcinoma and control group.

	EBV Detection				p value*
	Males		Females		
	Positive (%)	Negative (%)	Positive (%)	Negative (%)	
Nasopharyngeal carcinoma cases					
Paraffin blocks (n = 19)	9 (90.0)	1 (10.0)	7 (77.7)	2 (22.3)	0.466
Fresh tissue – biopsy (n = 17)	13 (100.0)	0 (0.0)	4 (100.0)	0 (0.0)	–
Peripheral blood (n=32)	13 (48.1)	14 (51.9)	4 (80.0)	1 (20.0)	0.190
Total (n=68)	35 (70.0)	15 (30.0)	15 (83.3)	3 (16.7)	0.272
Control Group					
Peripheral blood (n=45)	0 (0.0)	27 (100.0)	0 (0.0)	18 (100.0)	–

* Chi-square test

**Table 4:** EBV detection analysis according to the NPC tumoral stage

	EBV Detection		p value*
	EBV positive	EBV negative	
	N (%)	N (%)	
Tumoral stage			
Stages I/II/III	6 (40.0)	9 (60.0)	0.069
Stage IV	9 (75.0)	3 (25.0)	

* Chi-squared test

## DISCUSSION

The frequency of positive EBV cases in the tumor tissue samples in paraffin blocks was of 84.2%. Although the frequency of EBV positive cases is high, one could expect that all tissues would have EBV infected cells. This result is probably due to the DNA quality of the samples and efficacy of viral DNA extraction based on the samples of tumoral tissue fragments in paraffin blocks. Given the insufficient quality of the samples we used a nested PCR protocol, in an attempt to enhance the detection method sensitivity. In fact, the EBV detection results from the fresh tissue samples showed 100% of positive cases, corroborating other studies published which show that all the nasopharyngeal tumor cells had viral DNA^10,^^11^. In the case of samples from the peripheral blood in patients with NPC we found 53.1% of positive EBV cases. In the control group we did not find EBV DNA in the peripheral blood of the healthy individuals.

These results indicate a higher frequency of positive EBV cases among patients with NPC and such difference was statistically significant, regardless of the type of sample analyzed (P<0.001 for the paraffin block samples, fresh tissue and peripheral blood).

Recent studies indicate that patients with nasopharyngeal carcinoma have EBV DNA circulating in their peripheral blood^12^, ^13^, ^14^. The EBV detection analysis matched tumor stage (Table 4), indicating that the frequency of EBV-positive cases is higher in more advance stage tumors, although this difference is in the threshold of statistical significance (p=0.069). These results indicate that the most advanced tumors seem to release EBV DNA more easily or in greater quantity to the peripheral blood of diseased people.

EBV DNA was detected in all the samples from biopsies of nasopharyngeal fresh tumoral tissue, indicating that the PCR real-time protocol used is sensitive and indicated to detect EBV. The EBV detection analysis in the peripheral blood has been investigated in recent studies in order to assess the clinical implications of the EBV presence in the peripheral circulating blood from patients with nasopharyngeal carcinoma. This study indicates that the frequency of EBV-positive cases detected in the peripheral blood is higher in advanced studies. These results can serve as basis for future studies designed to assess the test value as an additional tumoral marker for diagnostic purposes. On the other hand, with the support of quantitative tests aiming at quantifying the levels of EBV in the blood of NPC patients, we will be able to assess the virus levels value as a prognostic marker in these patients. These tests seem to have a high medical value given the sensitiveness of the method and the ease of sampling, in which the only thing that is needed is to collect peripheral blood, a non-invasive technique, with a potential for clinical applicability.

The rates of nasopharyngeal carcinoma have a distinct distribution in ethnical and geographic terms. Given its low incidence in western countries, very little is known about the biology of these tumors in non-endemic areas, knowing that most of the studies are based in high risk population, with their own characteristics, which may not necessarily be the same seen in other countries. The unequal prevalence of these tumors throughout the world suggests a complex etiology associated with genetic and environmental factors, and both risk factors seem to be different in their geographic distribution, both in terms of environmental distribution and genetic background. Prior studies indicate that EBV needs a changed genetic environment in order to promote cell prolipheration7. The genetic profile can then alter the individual's susceptibility to cell immortalization by the virus, thus modulating the impact of the EBV effects on the carcinogenesis of these tumors.

## CONCLUSIONS

These results indicate that there are differences in the Epstein-Barr virus study in the group of patients with NPC and in the control group without tumor.

This study can help understand the biological mechanisms in the nasopharyngeal tumor and in the correlation of these tumors with the EBV infection in a non-endemic, low risk area.

## ACKNOWLEDGEMENT

This project was financed by the Ministry of Health of Portugal (CFICS – 261/1999).
